# Association analysis and exploratory mediation study of the stress-induced hyperglycaemia ratio and glycaemic ratio indices with diabetic retinopathy in patients with type 2 diabetes

**DOI:** 10.3389/fnut.2026.1798444

**Published:** 2026-05-08

**Authors:** Deyuan Zhang, Zhongyuan Jia, Anyi Geng, Zhihua Zhang, Xiaoyu Pan, Xing Zhong

**Affiliations:** 1 Department of Endocrinology, The Second Affiliated Hospital of Anhui Medical University, Hefei, Anhui, China; 2 Department of Epidemiology and Biostatistics, School of Public Health, Anhui Medical University, Hefei, Anhui, China

**Keywords:** diabetic retinopathy, fasting blood glucose/glycated hemoglobin, mediation effect, postprandial 2-h glucose/glycated hemoglobin, stress hyperglycaemia ratio, type 2 diabetes mellitus

## Abstract

**Objective:**

Diabetic retinopathy (DR) is a major microvascular complication of type 2 diabetes mellitus (T2DM), and traditional glycaemic control indicators struggle to comprehensively predict its risk. This study aims to investigate the association between the stress hyperglycaemia ratio (SHR), fasting blood glucose/glycated hemoglobin (FBG/HbA1c), postprandial 2-h glucose/glycated hemoglobin (P2hBG/HbA1c) with DR in T2DM patients, and the mediating effects of non-high-density lipoprotein cholesterol/high-density lipoprotein cholesterol ratio (NHHR) and triglycerides/high-density lipoprotein cholesterol ratio (TG/HDL-C).

**Methods:**

This study is a retrospective cross-sectional study based on inpatients at a single center. A total of 1,924 T2DM patients were enrolled and grouped according to DR status and quartiles of SHR, FBG/HbA1c, and P2hBG/HbA1c. Multivariate logistic regression, subgroup analysis, restricted cubic spline (RCS) analysis, and mediation effect analysis were employed to validate the associations between indicators and their underlying mechanisms.

**Results:**

The DR group exhibited a considerably higher proportion of participants in the upper quartiles for SHR, FBG/HbA1c and P2hBG/HbA1c. In the fully adjusted model (Model 4), the following comparisons were made between the Q1 quartile and the Q2, Q3 and Q4 quartiles: Q2 (OR = 1.65, 95% CI: 1.24–2.21), Q3 (OR = 1.75, 95% CI: 1.31–2.33) and Q4 (OR = 2.51, 95% CI: 1.88–3.36). For FBG/HbA1c, the following comparisons were made between Q2 (OR = 1.42, 95% CI: 1.06–1.91), Q3 (OR = 1.85, 95% CI: 1.39–2.48) and Q4 (OR = 2.95, 95% CI: 2.21–3.94). For P2hBG/HbA1c, the fourth quartile (OR = 1.56, 95% CI: 1.17–2.08). Subgroup analysis and RCS analysis confirmed a consistent linear association across all subgroups, with no significant non-linearity. The present study demonstrated that NHHR and TG/HDL-C exhibited significant independent mediating effects in the association between these glycaemic markers and DR.

**Conclusion:**

The present study found a positive correlation between SHR, FBG/HbA1c and P2hBG/HbA1c with the prevalence of DR in T2DM patients. Furthermore, NHHR and TG/HDL-C exhibited exploratory mediating effects in these associations.

## 1 Introduction

Diabetic retinopathy (DR) is the most disabling microvascular complication of type 2 diabetes mellitus (T2DM), affecting 34.6% of the global T2DM population, with 10.2% of cases classified as vision-threatening DR (1). Recent epidemiological data demonstrate that the global number of individuals afflicted with DR exceeded 103 million in 2021 and is projected to reach 160 million by 2045, thus classifying it as one of the most rapidly expanding causes of visual impairment on a global scale (2). As the foremost cause of irreversible blindness in working-age populations, DR significantly diminishes patients' quality of life and imposes a considerable socioeconomic and healthcare burden (3). Consequently, the identification of high-risk individuals and the implementation of targeted interventions are considered fundamental strategies to reduce the prevalence of blindness due to DR.

Conventionally, diabetes duration and glycated hemoglobin (HbA1c) are recognized as core risk factors for the onset and progression of DR (4). As the gold standard for reflecting average blood glucose over a period of 2–3 months, the dose-response relationship between HbA1c and DR has been verified in multiple large cohort studies (5, 6). Nevertheless, a persistent clinical paradox remains: approximately 20% of T2DM patients with long-term HbA1c within target levels still develop progressive DR (7, 8). A meta-analysis of multiple cohorts further confirmed that HbA1c has low sensitivity for predicting DR, indicating that a single indicator of long-term average glycaemia cannot fully capture the risk of DR onset (9). This discrepancy has prompted research into novel glycaemic metrics that capture short-term glycaemic variation and stress-related dysglycaemia, which are not fully reflected by HbA1c.

In recent years, mounting evidence has underscored the role of glycaemic fluctuations in the development of diabetic microvascular complications. The fasting blood glucose-to-HbA1c ratio (FBG/HbA1c) and the 2-h postprandial blood glucose-to-HbA1c ratio (P2hBG/HbA1c) serve as simplified indicators of glycaemic variability. By normalizing absolute glucose levels to HbA1c, these ratios have been shown to reduce confounding from long-term average glycaemia and better quantify the magnitude and frequency of short-term glucose excursions (10, 11). However, these ratios are predicated on routine daily glycaemic variation and do not account for dysglycaemia during physiological stress. The stress hyperglycaemia ratio (SHR) has emerged as a complementary metric that quantifies the relative elevation of glucose above basal levels during stress states such as inflammation, infection, or tissue injury. As stated in Wang et al. (12), the SHR is indicative of both the pancreatic β-cell reserve and the severity of insulin resistance under stress. Elevated SHR has been consistently linked to an increased risk of diabetic macrovascular complications and coronary heart disease (13–15). However, its association with microvascular complications, such as DR, remains understudied, and its mechanistic pathways are poorly defined.

Dyslipidaemia is a well-established pathophysiological contributor to diabetic complications, yet its role in DR remains controversial. Preliminary studies established a correlation between elevated total cholesterol (TC) and low-density lipoprotein cholesterol (LDL-C) and the risk of developing DR (16). However, subsequent meta-analyses have indicated that individual lipid markers possess limited predictive capability (17). Composite lipid indices, which integrate multiple lipid fractions, offer a more comprehensive measure of dyslipidaemia. Among these, the non-high-density lipoprotein to high-density lipoprotein cholesterol ratio (NHHR) is of particular interest as it simultaneously captures both atherogenic lipoprotein burden and anti-atherogenic high-density lipoprotein cholesterol (HDL-C) function. This enables more accurate risk stratification for microvascular disease than single lipid markers (18). The triglyceride-to-HDL-C ratio (TG/HDL-C) has been identified as a robust indicator of insulin resistance and has been demonstrated to be strongly associated with diabetic complications (19). Emerging evidence suggests that lipid metabolism may mediate the link between glycaemic stress and DR. However, prior research has focused on traditional glycaemic indicators and single lipid markers. To date, no study has systematically evaluated the combined associations of SHR, FBG/HbA1c, and P2hBG/HbA1c with DR, nor clarified whether NHHR and TG/HDL-C act as mediating pathways in these relationships.

To date, there have been few studies investigating the association between SHR and DR, and no research has simultaneously examined the relationship between SHR, the glycaemic variability ratio and DR, nor has the mediating role of composite lipid indices been clarified. The present study aims to investigate the association between the aforementioned indicators and DR in hospitalized patients with type 2 diabetes, and to analyse the exploratory mediating effects of NHHR and TG/HDL-C, thereby filling a gap in the research in this field.

## 2 Methods

### 2.1 Research design and subjects

**Figure 1:**
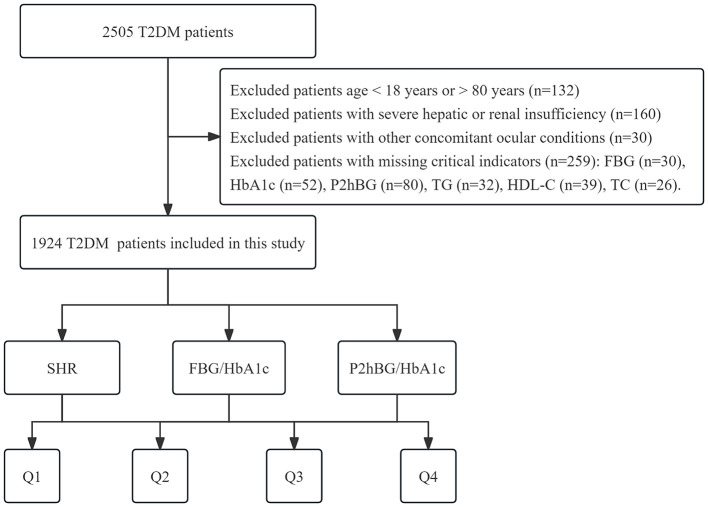
Flowchart of the population included in our study.

This retrospective cross-sectional study included patients with T2DM admitted to the Department of Endocrinology at the Second Affiliated Hospital of Anhui Medical University between January 2025 and December 2025. All inpatients meeting the criteria during the study period were consecutively enrolled. Inclusion criteria: (1) diagnosis of T2DM meeting the criteria outlined in the Chinese Guidelines for the Prevention and Treatment of Type 2 Diabetes (2020 Edition); (2) complete clinical records, including demographic characteristics, laboratory test results, and information on diagnosed complications. Exclusion criteria: (1) type 1 diabetes, gestational diabetes, or other secondary forms of diabetes; (2) concurrent ocular diseases; (3) severe hepatic or renal insufficiency, malignant tumors, acute infectious diseases, or autoimmune disorders; (4) missing key clinical indicators; (5) duplicate admissions were excluded using unique case numbers; (6) age < 18 years or >80 years. A total of 1,924 T2DM patients were ultimately included. This study was approved by the Medical Ethics Committee of the Second Affiliated Hospital of Anhui Medical University (Approval No: YX2024-210), and all patients provided written informed consent (Figure 1).

### 2.2 Grouping methods

The classification of DR was determined by two experienced ophthalmologists, who conducted dilated fundus examinations. The present study made reference to the diagnostic criteria outlined in the 2021 Edition of the Guidelines for the Prevention and Treatment of Diabetic Retinopathy. Patients were categorized into two groups: the diabetic retinopathy group (DR group, *n* = 686) and the non-diabetic retinopathy group (Non-DR group, *n* = 1,238). All study participants underwent color fundus photography, which was interpreted in a blinded manner by two experienced ophthalmologists in accordance with guideline standards, with the severity of DR recorded. In the event of a discrepancy between the two ophthalmologists' interpretations, a third senior ophthalmologist was consulted to ensure the standardization and reproducibility of the diagnosis. The quartile grouping method was employed to calculate the quartiles for SHR, FBG/HbA1c, and P2hBG/HbA1c. This entailed the division of the 1,924 patients into four subgroups, with each subgroup comprising 481 patients.

### 2.3 Data collection

A comprehensive data set should be collected from patients, encompassing their age, gender, the duration of diabetes, and their body mass index (BMI). Their systolic blood pressure (SBP) and diastolic blood pressure (DBP) should be measured. Comorbidities should be documented, including hypertension (HT), coronary heart disease (CHD), stroke, peripheral artery disease (PAD), and metabolic-associated steatotic liver disease (MASLD). The definitive diagnosis of all comorbidities was made by clinicians in accordance with the relevant disease management guidelines.

A fully automated biochemical analyser was employed to measure FBG, P2hBG, blood urea nitrogen (BUN), serum creatinine (Cr), uric acid (UA), TC, triglycerides (TG), LDL-C, high-density lipoprotein cholesterol (HDL-C), alanine aminotransferase (ALT), and aspartate aminotransferase (AST). HbA1c was measured by high-performance liquid chromatography. Additionally, white blood cell count (WBC), lymphocyte count, neutrophil count, hemoglobin (HGB), and platelet count (PLT) were also collected. Laboratory tests and DR fundus examinations for all study participants were conducted during the same hospital stay, with no more than 3 days elapsing between them.

Five milliliters of blood were collected from the antecubital vein of the study subjects in the morning on an empty stomach. The blood was subjected to a centrifugal process at a temperature of 4 °C and at a rotational velocity of 3,000 rpm for a duration of 10 min, with the objective of effecting the separation of the serum. The serum was subsequently stored at a temperature of −80 °C, pending analysis. Serum biochemical parameters were measured using a fully automated biochemical analyser (Cobas 8000, Roche Diagnostics GmbH, Germany). Prior to the commencement of the testing phase, the instrument was subjected to a rigorous metrological certification and calibration process using the manufacturer's designated calibration reagent (batch number 123456). The HbA1c level was measured using high-performance liquid chromatography (HPLC) with an HbA1c analyser (D-10, Bio-Rad Laboratories, USA) and the manufacturer's reagents (batch no. 789012). The wavelengths used were 415 nm and 690 nm, the mobile phase was pH 6.4 phosphate buffer, the flow rate was 1.0 mL/min, and the column temperature was 37 °C. The remaining serum biochemical parameters were measured using a fully automated biochemical analyser with reagents. The personnel conducting the tests had undergone specialist training to ensure the reliability of the results.

### 2.4 Indicator calculation

SHR, as a marker of stress-induced hyperglycaemia, has been shown to be associated with diabetic complications. In this study, it was used as an exposure variable to investigate the association between stress-related glycaemic abnormalities and diabetic retinopathy. SHR = FBG (mg/dL)/[(28.7 × HbA1c%) – 46.7]. FBG/HbA1c = FBG (mg/dL)/ HbA1c (%). P2hBG/HbA1c = P2hBG (mg/dL)/ HbA1c (%). TG/HDL-C = TG (mmol/L)/HDL-C (mmol/L). NHHR = [TC (mmol/L) – HDL-C (mmol/L)]/HDL-C (mmol/L).

### 2.5 Statistical analysis

The statistical analysis was conducted utilizing the R software version 4.2.1 (R 4.2.1 was developed by the R Core Team, with copyright owned by the R Foundation for Statistical Computing in Vienna, Austria). The Shapiro–Wilk test was used to assess normality, and parametric or non-parametric tests were selected for intergroup comparisons based on the results of this test. Data that is normally distributed is presented as the mean ± standard deviation (x ± s), with intergroup comparisons conducted using the independent samples *t*-test. Non-normally distributed data are presented as the median (interquartile range), with intergroup comparisons performed using the Mann–Whitney *U* test or Kruskal–Wallis *H* test. The count data were expressed as counts (percentages), with intergroup comparisons performed using the χ^2^ test or Fisher's exact probability test. Multicollinearity was assessed using the variance inflation factor (VIF), with a threshold of VIF < 10 to confirm that there was no significant multicollinearity among the variables; the goodness of fit of the logistic regression model was evaluated using the Hosmer–Lemeshow test, with a *P*-value > 0.05 indicating a good model fit. Multivariate logistic regression analysis was employed to investigate the independent associations between SHR, FBG/HbA1c, P2hBG/HbA1c, and the risk of DR occurrence. Four models were constructed, each adjusted in four steps: Model 1 was the unadjusted model; Model 2 adjusted for demographic characteristics (age, gender, and diabetes duration); Model 3 further adjusted for CHD, stroke, HT, PAD, and MASLD; and Model 4 supplemented Model 3 by adjusting for ALT, AST, BUN, Cr, and UA. The strength of the association was expressed as odds ratios (OR) with 95% confidence intervals (CI). The inclusion of confounding factors is based on both the existing literature and clinical experience.

Subgroup analyses were conducted to investigate whether the association between SHR, FBG/HbA1c, P2hBG/HbA1c, and DR differed across subgroups defined by age, sex, BMI, HT, CHD, stroke, PAD, and MASLD. Interaction tests were performed to assess heterogeneity of effects across subgroups. Restricted cubic spline (RCS) analysis was employed to investigate the non-linear relationship between SHR, FBG/HbA1c, P2hBG/HbA1c, and DR, adjusting for relevant confounding factors. In the RCS analysis, four nodes were defined, with their positions determined based on the characteristics of the data distribution and professional expertise, in order to accurately fit the dose-response relationship between the variables and the outcome measures. The identification of outliers was conducted through the utilization of the absolute deviation method. Subsequent to the verification process, outliers were excluded with the objective of preventing the distortion of statistical outcomes. The mediation analysis was performed using the mediation package in R in order to investigate the mediating roles of NHHR and TG/HDL-C in the relationships between SHR, FBG/HbA1c, P2hBG/HbA1c, and DR. The binary outcomes were analyzed using logistic regression models. Bootstrap sampling (*n* = 1,000) was employed to estimate the 95% CI for the mediation effects, and direct, indirect and total effects were calculated to ensure methodological transparency and reproducibility. All statistical tests were two-tailed, with *P* < 0.05 indicating statistically significant differences.

## 3 Results

### 3.1 Demographic and baseline characteristics of T2DM patients based on DR grouping

The present study comprised a total of 1,924 patients with T2DM, of whom 1,238 were in the Non-DR group and 686 in the DR group. Baseline comparisons revealed older age and longer diabetes duration in the DR group (*P* < 0.001), with no significant gender distribution difference (*P* = 0.051). With regard to blood pressure, the DR group demonstrated higher SBP and lower DBP (*P* < 0.05), while BMI was marginally lower than in the Non-DR group (*P* = 0.029). Among glucose-related indicators, the DR group exhibited significantly higher proportions in the upper quartile for FBG, P2hBG, FBG/HbA1c, and P2hBG/HbA1c (*P* < 0.001), though HbA1c levels were marginally lower (*P* = 0.003). With regard to the analysis of lipid profiles, the DR group demonstrated significantly lower levels of TC, LDL-C, and TG in comparison to the Non-DR group (*P* < 0.05), with no observed intergroup variation in HDL-C (*P* = 0.172). In the context of complete blood counts, the DR group exhibited significantly diminished lymphocyte counts and hemoglobin levels (*P* < 0.001), with no statistically significant differences observed in other parameters (*P* > 0.05). Liver and kidney function indicators revealed higher blood urea nitrogen levels in the DR group (*P* < 0.001) and lower ALT and AST levels (both *P* < 0.001). With regard to comorbidities, the DR group demonstrated a significantly higher prevalence of CHD, stroke, and MASLD (*P* < 0.001), while exhibiting a lower prevalence of HT and PAD (*P* < 0.05). Furthermore, the DR group exhibited a higher proportion of SHR in the upper quartile (*P* < 0.001). These observations suggest a potential association between these baseline characteristics and the development and progression of DR (Table 1).

**Table 1:** Demographic and baseline characteristics of T2DM patients based on DR grouping.

Variables	Total (*n* = 1,924)	Non-DR (*n* = 1,238)	DR (*n* = 686)	*P-*value
Age (y)	54.48 ± 13.65	53.55 ± 14.36	56.16 ± 12.10	<0.001
Duration (y)	6.00 (1.00, 10.00)	4.00 (0.50, 10.00)	10.00 (4.00, 15.00)	<0.001
BMI (kg/m^2^)	25.55 ± 3.56	25.68 ± 3.54	25.31 ± 3.58	0.029
SBP (mmHg)	132.57 ± 17.47	131.58 ± 16.96	134.36 ± 18.23	0.001
DBP (mmHg)	80.29 ± 12.45	80.80 ± 12.23	79.38 ± 12.78	0.017
FBG (mmol/L)	7.80 ± 2.62	7.46 ± 2.36	8.41 ± 2.95	<0.001
P2hBG (mmol/L)	16.40 ± 4.61	16.10 ± 4.43	16.93 ± 4.88	<0.001
HbA1c (%)	9.35 ± 2.18	9.46 ± 2.22	9.16 ± 2.10	0.003
BUN (mmol/L)	6.56 ± 3.70	6.18 ± 2.84	7.26 ± 4.81	<0.001
UA (mmol/L)	323.00 (262.00, 392.00)	323.00 (263.00, 395.75)	323.00 (261.00, 383.00)	0.534
TC (mmol/L)	4.75 (3.99, 5.52)	4.79 (4.09, 5.56)	4.66 (3.82, 5.45)	0.002
HDL-C (mmol/L)	1.15 ± 0.35	1.14 ± 0.37	1.16 ± 0.31	0.172
LDL-C (mmol/L)	2.91 ± 0.88	2.94 ± 0.89	2.85 ± 0.86	0.022
TG (mmol/L)	1.55 (1.07, 2.45)	1.60 (1.10, 2.61)	1.41 (0.99, 2.19)	<0.001
WBC ( × 10^9^/L)	6.77 ± 4.42	6.81 ± 4.60	6.70 ± 4.08	0.601
Neutrophil ( × 10^9^/L)	3.80 ± 1.90	3.79 ± 1.88	3.81 ± 1.92	0.746
Lymphocyte ( × 10^9^/L)	2.05 ± 0.73	2.10 ± 0.73	1.96 ± 0.72	<0.001
HGB (g/L)	137.19 ± 18.01	139.12 ± 17.49	133.72 ± 18.42	<0.001
PLT ( × 10^9^/L)	200.13 ± 60.11	200.69 ± 59.76	199.12 ± 60.76	0.583
ALT (U/L)	21.00 (15.00, 33.00)	22.00 (15.00, 36.00)	19.00 (14.00, 28.00)	<0.001
AST (U/L)	20.00 (17.00, 27.00)	21.00 (17.00, 28.00)	20.00 (16.00, 25.00)	<0.001
Creatine (mmol/L)	60.50 (50.00, 73.00)	60.00 (50.00, 72.00)	61.00 (50.00, 76.00)	0.111
Gender (male), *n* (%)	1,258 (65.38)	829 (66.96)	429 (62.54)	0.051
CHD, *n* (%)	86 (4.47)	40 (3.23)	46 (6.71)	<0.001
Stroke, *n* (%)	207 (10.76)	109 (8.80)	98 (14.29)	<0.001
HT, *n* (%)	1,114 (57.90)	750 (60.58)	364 (53.06)	0.001
PAD, *n* (%)	468 (24.32)	339 (27.38)	129 (18.80)	<0.001
MASLD, *n* (%)	998 (51.87)	586 (47.33)	412 (60.06)	<0.001
SHR, *n* (%)				< 0.001
Q1	481 (25.00)	366 (29.56)	115 (16.76)	
Q2	481 (25.00)	318 (25.69)	163 (23.76)	
Q3	481 (25.00)	303 (24.47)	178 (25.95)	
Q4	481 (25.00)	251 (20.27)	230 (33.53)	
FBG/HbA1c, *n* (%)				< 0.001
Q1	481 (25.00)	368 (29.73)	113 (16.47)	
Q2	481 (25.00)	331 (26.74)	150 (21.87)	
Q3	481 (25.00)	301 (24.31)	180 (26.24)	
Q4	481 (25.00)	238 (19.22)	243 (35.42)	
P2hBG/HbA1c, *n* (%)				< 0.001
Q1	481 (25.00)	342 (27.63)	139 (20.26)	
Q2	481 (25.00)	319 (25.77)	162 (23.62)	
Q3	481 (25.00)	303 (24.47)	178 (25.95)	
Q4	481 (25.00)	274 (22.13)	207 (30.17)	
NHHR	3.42 ± 1.65	3.51 ± 1.44	3.25 ± 1.95	<0.001
TG/HDL-C	2.32 ± 3.18	2.48 ± 3.37	2.03 ± 2.79	0.002

BMI, body mass index; SBP, systolic blood pressure; DBP, diastolic blood pressure; P2hBG, Postprandial 2-h blood glucose; HbA1c, glycated hemoglobin; WBC, white blood cell count; PLT, blood platelet count; BUN, blood urea nitrogen; Scr, scrum creatinine; UA, uric acid; TC, total cholesterol; TG, triglyceride; HDL-C, high-density lipoprotein cholesterol; LDL-C, low-density lipoprotein cholesterol; FBG, fasting blood glucose; HGB, hemoglobin; ALT, alanine aminotransferase; AST, aspartate transaminase; CHD, coronary heart disease; HT, hypertension; PAD, peripheral arterial disease; MASLD, metabolic dysfunction-associated steatotic liver disease; DR, diabetic retinopathy; NHHR, non-high-density lipoprotein to high-density lipoprotein cholesterol ratio.

### 3.2 Demographic and baseline characteristics of T2DM patients based on SHR, FBG/HbA1c and P2hBG/HbA1c grouping

The 1,924 subjects were divided into four groups (*n* = 481 per group) based on quartiles of FBG/HbA1c, P2hBG/HbA1c, and SHR. The investigation revealed that with increasing quartiles of the three grouping indicators, age and disease duration exhibited a marked increase (all *P* < 0.001), while BMI demonstrated a significant decrease (all *P* < 0.001). Furthermore, DBP exhibited a notable decrease (all *P* < 0.001), with no significant intergroup differences observed in SBP (*P* = 0.542, 0.098, 0.916, respectively for the three groups). Additionally, FBG, FBG/HbA1c, P2hBG/HbA1c, and SHR exhibited a significant increase (all *P* < 0.001), while HbA1c demonstrated a significant decrease (all *P* < 0.001). Moreover, BUN levels increased (*P* = 0.003, 0.007, < 0.001, respectively across the three groups), while TC and LDL-C levels decreased (all *P* < 0.001), and UA levels increased. A statistically significant decrease was observed only in the P2hBG/HbA1c subgroup (*P* < 0.001). No statistically significant differences were observed in the other two subgroups (*P* = 0.130 and 0.154, respectively). No significant intergroup differences were observed for HDL-C, TG, or creatinine (all *P* > 0.05); however, lymphocyte count, HGB, and PLT were significantly reduced (all *P* < 0.001). The WBC demonstrated a significant difference exclusively within the P2hBG/HbA1c subgroup (*P* = 0.021), while the neutrophil count exhibited no consistent significant variation (all *P* > 0.05). A statistically significant decrease was observed in both ALT and AST (*P* < 0.001). The proportion of males, and the prevalence of HT and PAD, were significantly lower (all *P* < 0.05). The prevalence of MASLD and DR was found to be significantly higher in T2DM patients (*P* < 0.001). There were no significant intergroup differences in the prevalence of CHD or stroke (all *P* > 0.05; Table 2, Supplementary Tables S1, S2).

**Table 2:** Participants demographics and baseline characteristics based on SHR quartiles.

Variables	SHR				*P-*value
	Q1 (*n* = 481)	Q2 (*n* = 481)	Q3 (*n* = 481)	Q4 (*n* = 481)	
Age (y)	50.95 ± 15.38	52.52 ± 13.86	56.12 ± 12.22	58.32 ± 11.61	<0.001
Duration (y)	2.00 (0.10, 8.00)	5.00 (1.00, 10.00)	9.00 (3.00, 11.00)	10.00 (4.00, 14.00)	<0.001
BMI (kg/m^2^)	26.31 ± 3.83	25.86 ± 3.59	25.13 ± 3.28	24.89 ± 3.32	<0.001
SBP (mmHg)	132.20 ± 17.13	132.46 ± 17.13	132.65 ± 17.85	132.98 ± 17.80	0.916
DBP (mmHg)	81.83 ± 12.32	81.55 ± 12.41	79.61 ± 12.98	78.19 ± 11.73	<0.001
FBG (mmol/L)	6.04 ± 1.46	7.37 ± 1.79	7.94 ± 2.11	9.84 ± 3.21	<0.001
HbA1c (%)	10.94 ± 1.94	9.72 ± 1.99	8.68 ± 1.87	8.08 ± 1.75	<0.001
FBG/HbA1c	9.93 ± 1.55	13.56 ± 1.03	16.33 ± 1.24	21.71 ± 4.17	<0.001
P2hBG/HbA1c	24.59 ± 7.28	30.11 ± 7.54	35.02 ± 8.11	41.13 ± 9.47	<0.001
BUN (mmol/L)	6.23 ± 4.64	6.37 ± 2.57	6.50 ± 2.59	7.15 ± 4.42	<0.001
UA (mmol/L)	331.00 (262.00, 410.00)	323.00 (267.00, 392.00)	321.00 (259.00, 386.00)	319.00 (259.00, 382.00)	0.154
TC (mmol/L)	4.83 (4.25, 5.65)	4.82 (4.16, 5.60)	4.75 (3.94, 5.55)	4.45 (3.71, 5.27)	<0.001
HDL-C (mmol/L)	1.13 ± 0.30	1.14 ± 0.36	1.17 ± 0.31	1.16 ± 0.41	0.160
LDL-C (mmol/L)	3.04 ± 0.94	3.00 ± 0.89	2.88 ± 0.84	2.72 ± 0.82	<0.001
TG (mmol/L)	1.56 (1.10, 2.59)	1.60 (1.16, 2.62)	1.53 (1.04, 2.39)	1.50 (0.96, 2.34)	0.014
WBC ( × 10^9^/L)	6.89 ± 3.68	6.93 ± 5.25	6.56 ± 3.53	6.70 ± 4.97	0.539
Neutrophil ( × 10^9^/L)	3.92 ± 2.32	3.71 ± 1.42	3.76 ± 1.98	3.79 ± 1.77	0.386
Lymphocyte ( × 10^9^/L)	2.22 ± 0.80	2.12 ± 0.70	1.99 ± 0.69	1.86 ± 0.67	<0.001
HGB (g/L)	141.18 ± 17.23	140.67 ± 16.85	135.16 ± 16.75	131.76 ± 19.39	<0.001
PLT ( × 10^9^/L)	208.18 ± 58.97	198.55 ± 53.98	201.31 ± 65.86	192.48 ± 60.17	<0.001
ALT (U/L)	24.00 (16.00, 37.00)	23.00 (16.00, 36.00)	20.00 (14.00, 29.00)	19.00 (13.00, 27.00)	<0.001
AST (U/L)	21.00 (18.00, 28.00)	20.00 (17.00, 28.00)	20.00 (16.00, 26.00)	19.00 (16.00, 24.00)	<0.001
Creatine (mmol/L)	60.00 (51.00, 72.00)	61.00 (52.00, 72.00)	60.00 (50.00, 71.00)	61.00 (49.00, 75.00)	0.514
Gender (male), *n* (%)	341 (70.89)	341 (70.89)	296 (61.54)	280 (58.21)	<0.001
CHD, *n* (%)	18 (3.74)	15 (3.12)	23 (4.78)	30 (6.24)	0.099
Stroke, *n* (%)	45 (9.36)	46 (9.56)	54 (11.23)	62 (12.89)	0.252
HT, *n* (%)	309 (64.24)	288 (59.88)	268 (55.72)	249 (51.77)	<0.001
PAD, *n* (%)	163 (33.89)	122 (25.36)	110 (22.87)	73 (15.18)	<0.001
MASLD, *n* (%)	223 (46.36)	215 (44.70)	269 (55.93)	291 (60.50)	<0.001
DR, *n* (%)	115 (23.91)	163 (33.89)	178 (37.01)	230 (47.82)	<0.001

BMI, body mass index; SBP, systolic blood pressure; DBP, diastolic blood pressure; P2hBG, Postprandial 2-h blood glucose; HbA1c, glycated hemoglobin; WBC, white blood cell count; PLT, blood platelet count; BUN, blood urea nitrogen; Scr, scrum creatinine; UA, uric acid; TC, total cholesterol; TG, triglyceride; HDL-C, high-density lipoprotein cholesterol; LDL-C, low-density lipoprotein cholesterol; FBG, fasting blood glucose; HGB, hemoglobin; ALT, alanine aminotransferase; AST, aspartate transaminase; CHD, coronary heart disease; HT, Hypertension; PAD; Peripheral arterial disease; MASLD, Metabolic dysfunction-associated steatotic liver disease; DR, diabetic retinopathy.

### 3.3 Multivariate logistic regression analyses

Multivariate logistic regression analysis revealed that compared with the SHR Q1 group, the risk of DR was significantly elevated in the Q2, Q3, and Q4 groups (all *P* < 0.001). In Model 1, OR = 1.63 (95% CI 1.23–2.16) for Q2, OR = 1.87 (95% CI 1.41–2.47) for Q3, and OR = 2.92 (95% CI 2.21–3.84) for Q4; In Model 2, OR = 1.61 (95% CI 1.22–2.14) for Q2, OR = 1.78 (95% CI 1.34–2.36) for Q3, OR = 2.72 (95% CI 2.06–3.60) for Q4; In Model 3, OR = 1.63 (95% CI 1.23–2.17) for Q2, OR = 1.76 (95% CI 1.32–2.33) for Q3, OR = 2.62 (95% CI 1.97–3.48) for Q4; In Model 4, OR = 1.65 (95% CI 1.24–2.21) for Q2, OR = 1.75 (95% CI 1.31–2.33) for Q3, and OR = 2.51 (95% CI 1.88–3.36) for Q4, indicating a positive correlation between SHR levels and DR risk (Table 3).

**Table 3:** Multivariate logistic regression analyses of SHR and diabetic retinopathy.

Variables	Model1		Model2		Model3		Model4	
	OR (95%CI)	*P-*value	OR (95%CI)	*P-*value	OR (95%CI)	*P-*value	OR (95%CI)	*P-*value
SHR								
Q1	1.00 (Reference)		1.00 (Reference)		1.00 (Reference)		1.00 (Reference)	
Q2	1.63 (1.23–2.16)	<0.001	1.61 (1.22–2.14)	<0.001	1.63 (1.23–2.17)	<0.001	1.65 (1.24–2.21)	<0.001
Q3	1.87 (1.41–2.47)	<0.001	1.78 (1.34–2.36)	<0.001	1.76 (1.32–2.33)	<0.001	1.75 (1.31–2.33)	<0.001
Q4	2.92 (2.21–3.84)	<0.001	2.72 (2.06–3.60)	<0.001	2.62 (1.97–3.48)	<0.001	2.51 (1.88–3.36)	<0.001

Model1: Crude; Model2: Adjust: Gender, Duration, BMI, and Age; Model3: Adjust: Gender, Duration, BMI, CHD, Stroke, HT, PAD, MASLD and Age; Model3: Adjust: Gender, Duration, BMI, CHD, Stroke, HT, Age, ALT, AST, BUN, Cr, and UA.

Compared with the FBG/HbA1c Q1 group, the risk of DR was significantly elevated in the Q2, Q3, and Q4 groups. In Model 1, OR = 1.48 (95% CI 1.11–1.96, *P* = 0.008) for Q2, OR = 1.95 (95% CI 1.47–2.58, *P* < 0.001) for Q3; OR = 3.33 (95% CI 2.52–4.38, *P* < 0.001) for Q4; In Model 2, OR = 1.44 (95% CI 1.08–1.92, *P* = 0.012) for Q2, OR = 1.86 (95% CI 1.40–2.47, *P* < 0.001) for Q3, OR = 3.13 (95% CI 2.37–4.14, *P* < 0.001) for Q4; in Model 3, OR = 1.42 (95% CI 1.06–1.90, *P* = 0.018) for Q2, OR = 1.87 (95% CI 1.40–2.49, *P* < 0.001) for Q3, OR = 3.04 (95% CI 2.29–4.03, *P* < 0.001) for Q4; In Model 4, OR = 1.42 (95% CI 1.06–1.91, *P* = 0.018) for Q2, OR = 1.85 (95% CI 1.39–2.48, *P* < 0.001) for Q3, OR = 2.95 (95% CI 2.21–3.94, *P* < 0.001) for Q4, indicating a positive correlation between FBG/HbA1c levels and DR risk (Table 4).

**Table 4:** Multivariate logistic regression analyses of FBG/HbA1c and diabetic retinopathy.

Variables	Model1		Model2		Model3		Model4	
	OR (95%CI)	*P-*value	OR (95%CI)	*P-*value	OR (95%CI)	*P-*value	OR (95%CI)	*P-*value
FBG/HbA1c								
Q1	1.00 (Reference)		1.00 (Reference)		1.00 (Reference)		1.00 (Reference)	
Q2	1.48 (1.11–1.96)	0.008	1.44 (1.08–1.92)	0.012	1.42 (1.06–1.90)	0.018	1.42 (1.06–1.91)	0.018
Q3	1.95 (1.47–2.58)	<0.001	1.86 (1.40–2.47)	<0.001	1.87 (1.40–2.49)	<0.001	1.85 (1.39–2.48)	<0.001
Q4	3.33 (2.52–4.38)	<0.001	3.13 (2.37–4.14)	<0.001	3.04 (2.29–4.03)	<0.001	2.95 (2.21–3.94)	<0.001

Model1: Crude; Model2: Adjust: Gender, Duration, BMI, and Age; Model3: Adjust: Gender, Duration, BMI, CHD, Stroke, HT, PAD, MASLD, and Age; Model3: Adjust: Gender, Duration, BMI, CHD, Stroke, HT, Age, ALT, AST, BUN, Cr, and UA.

Compared with the P2hBG/HbA1c Q1 group, the incidence of DR was significantly higher in the Q3 and Q4 groups, whilst the Q2 group showed no statistically significant association. In Model 1, the Q2 group had an OR = 1.25 (95% CI 0.95–1.64, *P* = 0.110), OR = 1.45 (95% CI 1.10–1.89, *P* = 0.008) for Q3, and OR = 1.86 (95% CI 1.42–2.43, *P* < 0.001) for Q4; In Model 2, Q2 group OR = 1.22 (95% CI 0.93–1.61, *P* = 0.152), Q3 group OR = 1.35 (95% CI 1.03–1.78, *P* = 0.032), Q4 group OR = 1.69 (95% CI 1.28–2.22, *P* < 0.001); In Model 3, Q2 group OR = 1.20 (95% CI 0.91–1.58, *P* = 0.208), Q3 group OR = 1.30 (95% CI 0.99–1.72, *P* = 0.062), Q4 group OR = 1.60 (95% CI 1.21–2.11, *P* < 0.001); In Model 4, Q2 group OR = 1.20 (95% CI 0.90–1.60, *P* = 0.204), Q3 group OR = 1.27 (95% CI 0.95–1.69, *P* = 0.100), Q4 group OR = 1.56 (95% CI 1.17–2.08, *P* = 0.003), indicating a positive correlation between P2hBG/HbA1c levels and DR risk. The increased risk in the Q4 group remained statistically significant after adjustment for multiple confounding factors (Table 5).

**Table 5:** Multivariate logistic regression analyses of P2hBG/HbA1c and diabetic retinopathy.

Variables	Model1		Model2		Model3		Model4	
	OR (95%CI)	*P-*value	OR (95%CI)	*P-*value	OR (95%CI)	*P-*value	OR (95%CI)	*P-*value
FBG/HbA1c								
Q1	1.00 (Reference)		1.00 (Reference)		1.00 (Reference)		1.00 (Reference)	
Q2	1.25 (0.95–1.64)	0.110	1.22 (0.93–1.61)	0.152	1.20 (0.91–1.58)	0.208	1.20 (0.90–1.60)	0.204
Q3	1.45 (1.10–1.89)	0.008	1.35 (1.03–1.78)	0.032	1.30 (0.99–1.72)	0.062	1.27 (0.95–1.69)	0.100
Q4	1.86 (1.42–2.43)	<0.001	1.69 (1.28–2.22)	<0.001	1.60 (1.21–2.11)	<0.001	1.56 (1.17–2.08)	0.003

Model1: Crude; Model2: Adjust: Gender, Duration, BMI, and Age; Model3: Adjust: Gender, Duration, BMI, CHD, Stroke, HT, PAD, MASLD and Age; Model3: Adjust: Gender, Duration, BMI, CHD, Stroke, HT, Age, ALT, AST, BUN, Cr, and UA.

### 3.4 Subgroup analyses

**Figure 2:**
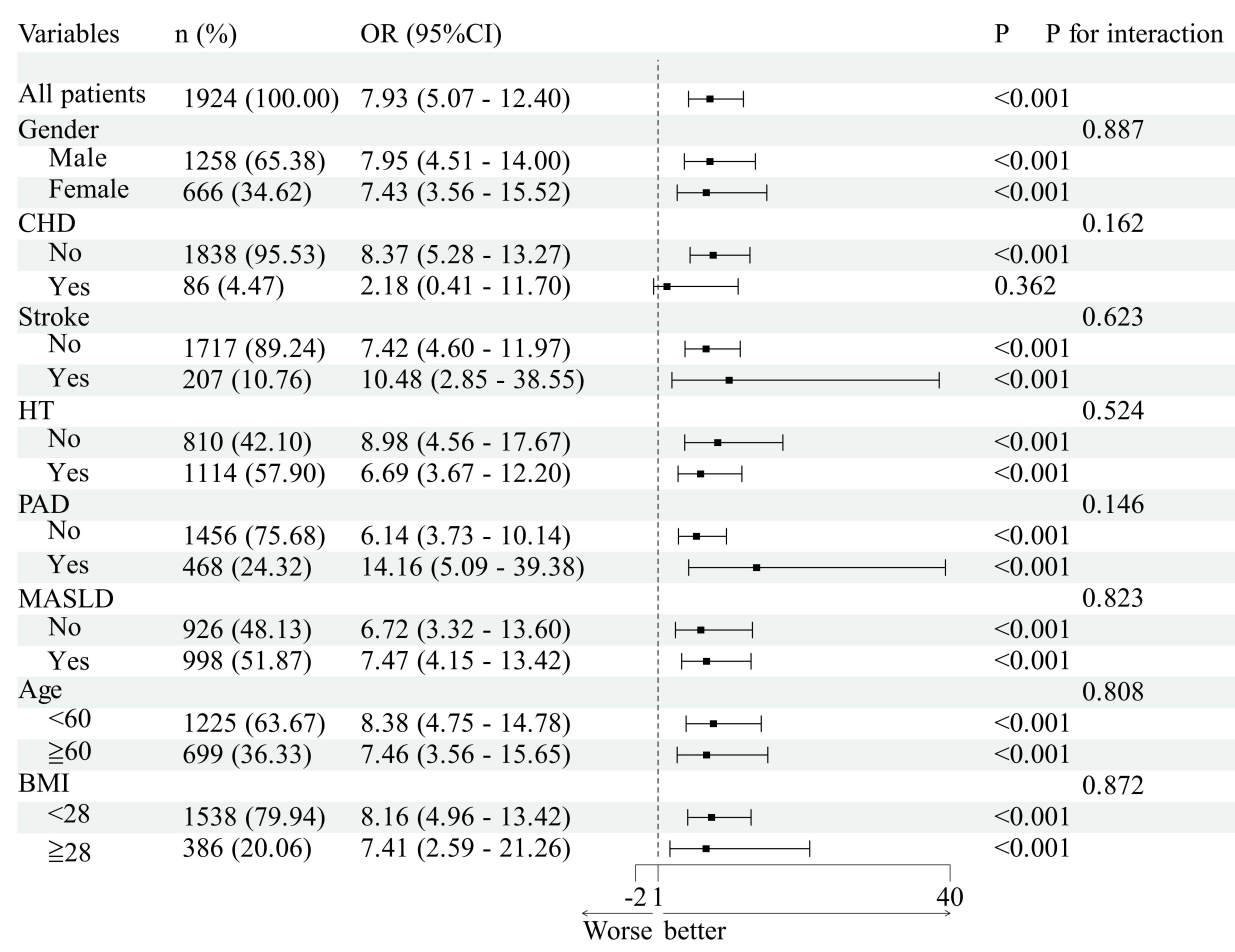
Subgroup analyses of the association between SHR and DR in patients with T2DM. The OR values shown in the figure are fully adjusted odds ratios comparing each quartile with the lowest quartile (Q1); 95% CI denotes the confidence interval; and *P* values refer to those from interaction or association tests.

**Figure 3:**
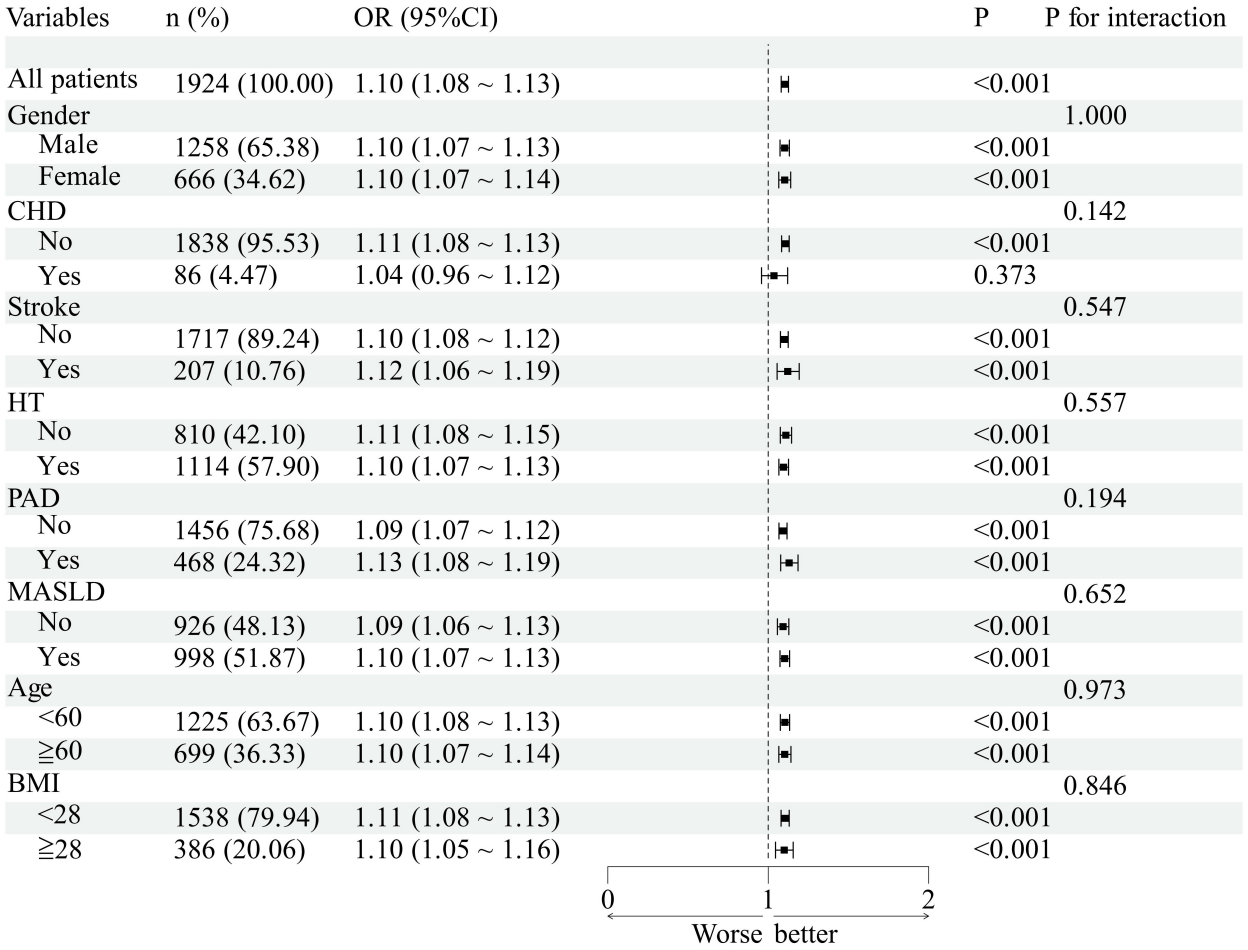
Subgroup analyses of the association between FBG/HbA1c and DR in patients with T2DM. The OR values shown in the figure are fully adjusted odds ratios comparing each quartile with the lowest quartile (Q1); 95% CI denotes the confidence interval; and *P* values refer to those from interaction or association tests.

**Figure 4:**
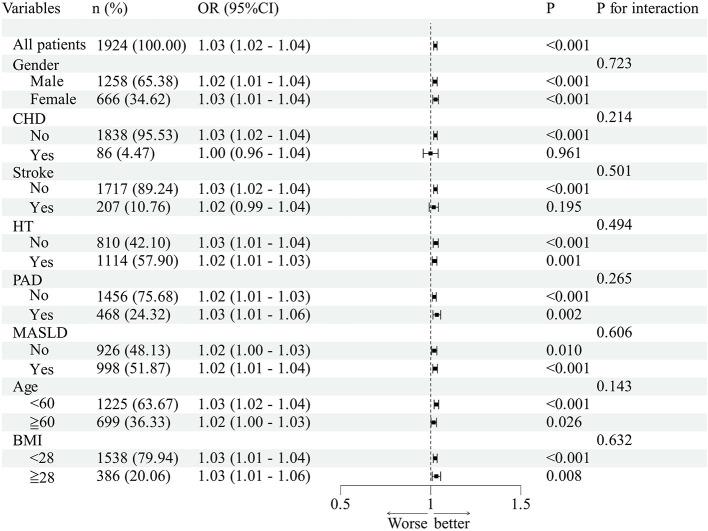
Subgroup analyses of the association between P2hBG/HbA1c and DR in patients with T2DM. The OR values shown in the figure are fully adjusted odds ratios comparing each quartile with the lowest quartile (Q1); 95% CI denotes the confidence interval; and *P* values refer to those from interaction or association tests.

The association between SHR, FBG/HbA1c and P2hBG/HbA1c with DR prevalence remained consistent across age, sex, BMI, hypertension, CHD, stroke, PAD and MASLD subgroups (Figures 2–4, all *P* for interaction >0.05).

### 3.5 Non-linear relationship analysis

**Figure 5:**
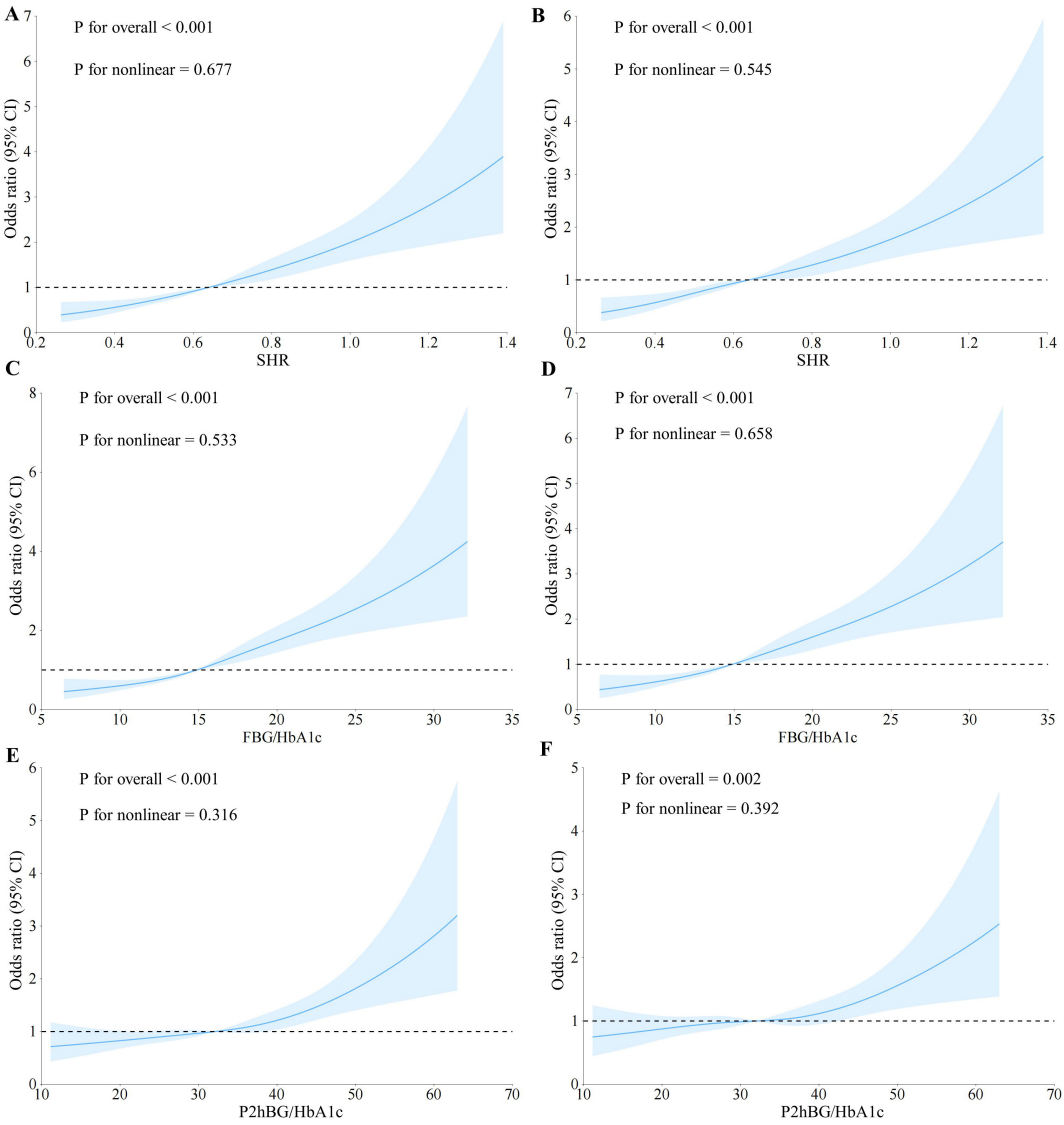
Results of non-linear correlation analysis. **(A)** Non-linear correlation analysis between SHR and DR, unadjusted for confounding factors. **(B)** Non-linear correlation analysis between SHR and DR, adjusting for confounding factors. **(C)** Non-linear correlation analysis between FBG/HbA1c and DR, unadjusted for confounding factors. **(D)** Non-linear correlation analysis between FBG/HbA1c and DR, adjusting for confounding factors. **(E)** Non-linear correlation analysis between P2hBG/HbA1c and DR, unadjusted for confounding factors. **(F)** Non-linear correlation analysis between P2hBG/HbA1c and DR, adjusting for confounding factors. The adjusted confounding factors include: Gender, Duration, BMI, CHD, Stroke, HT, Age, ALT, AST, BUN, Cr, UA, TC, TG, HDL-c, and LDL-c.

In order to further investigate the non-linear relationship between SHR, FBG/HbA1c, and P2hBG/HbA1c with DR in T2DM patients, RCS analysis was conducted. The results of the RCS indicated an absence of a significant non-linear association between SHR, FBG/HbA1c, and P2hBG/HbA1c with DR prevalence, even after adjusting for several confounding factors (Figure 5).

### 3.6 Exploratory statistical analysis of lipid metabolites

**Figure 6:**
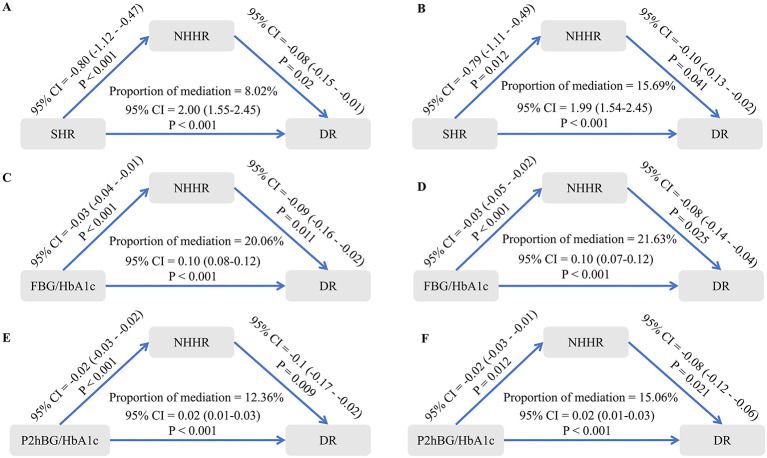
The mediating effect of NHHR on the relationship between SHR, FBG/HbA1c, P2hBG/HbA1c, and DR. **(A)** The mediating effect of NHHR on the relationship between SHR and DR, unadjusted for confounding factors. **(B)** The mediating effect of NHHR on the relationship between SHR and DR, adjusting for confounding factors. **(C)** The mediating effect of NHHR on the relationship between FBG/HbA1c and DR, unadjusted for confounding factors. **(D)** The mediating effect of NHHR on the relationship between FBG/HbA1c and DR, adjusting for confounding factors. **(E)** The mediating effect of NHHR on the relationship between P2hBG/HbA1c and DR, unadjusted for confounding factors. **(F)** The mediating effect of NHHR on the relationship between P2hBG/HbA1c and DR, adjusting for confounding factors.

**Figure 7:**
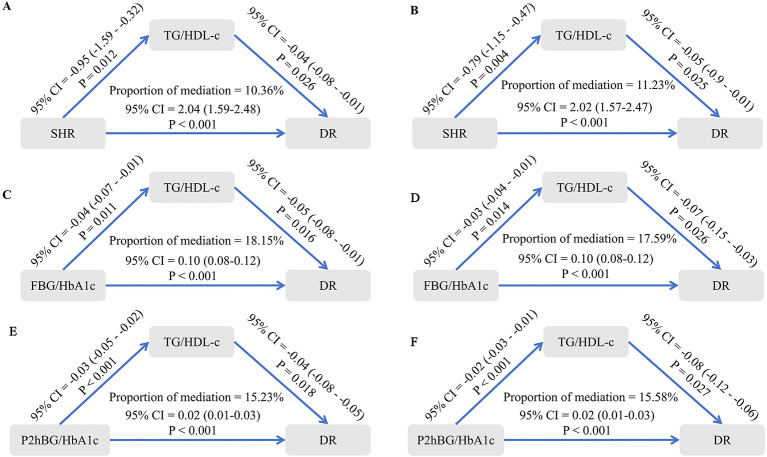
The mediating effect of TG/HDL-C on the relationship between SHR, FBG/HbA1c, P2hBG/HbA1c, and DR. **(A)** The mediating effect of TG/HDL-C on the relationship between SHR and DR, unadjusted for confounding factors. **(B)** The mediating effect of TG/HDL-C on the relationship between SHR and DR, adjusting for confounding factors. **(C)** The mediating effect of TG/HDL-C on the relationship between FBG/HbA1c and DR, unadjusted for confounding factors. **(D)** The mediating effect of TG/HDL-C on the relationship between FBG/HbA1c and DR, adjusting for confounding factors. **(E)** The mediating effect of TG/HDL-C on the relationship between P2hBG/HbA1c and DR, unadjusted for confounding factors. **(F)** The mediating effect of TG/HDL-C on the relationship between P2hBG/HbA1c and DR, adjusting for confounding factors.

NHHR and TG/HDL-C exerted partial statistical mediating effects on the associations between SHR, FBG/HbA1c, P2hBG/HbA1c, and DR, which persisted even after adjusting for multiple confounding factors (all *P* < 0.05; Figures 6, 7).

## 4 Discussion

This study systematically revealed, through large-scale cross-sectional analysis, the positive correlation between SHR, FBG/HbA1c, P2hBG/HbA1c and the risk of DR occurrence in T2DM patients. The study also elucidated the key mediating role played by the composite lipid markers NHHR and TG/HDL-C in the relationship between the aforementioned blood glucose-related markers and diabetic retinopathy. These findings not only deepen our understanding of the risk factors for DR, but also provide new insights into the molecular mechanisms underlying the association between abnormal blood glucose levels and DR.

This study found that patients in the DR group were older and had a longer duration of diabetes, consistent with previous epidemiological findings (20). Advancing age leads to functional decline in retinal microvascular endothelial cells and diminished oxidative stress clearance capacity, whilst prolonged diabetes causes sustained damage to retinal microvessels through pathways including advanced glycation end-product (AGEs) accumulation and inflammatory response activation (21). A study based on the Chinese population demonstrated that each additional year of diabetes duration increases the risk of DR by 6.1%, highly consistent with the baseline disparity trend observed in this study (22).

With regard to glycaemic indicators, the study observed a noteworthy phenomenon: patients in the DR group had higher FBG and P2hBG levels, yet slightly lower HbA1c levels. This may be related to the more intensive glycaemic control treatment administered to DR patients. Furthermore, the proportion of patients in the highest quartile for SHR, FBG/HbA1c and P2hBG/HbA1c was significantly higher. The elevated ratios of FBG/HbA1c and P2hBG/HbA1c, serving as simplified indicators of glycaemic fluctuation, reflect pronounced short-term blood glucose oscillations. As demonstrated in previous studies, fluctuations in blood glucose levels have been shown to promote retinal neovascularisation by influencing the expression of vascular endothelial growth factor (VEGF) (23, 24). As demonstrated in the following *in vitro* study, fluctuations in blood glucose levels have been shown to significantly increase the permeability of retinal microvascular endothelial cells by activating the VEGF pathway (25). Moreover, extant research has demonstrated that elevated SHRs can exert a detrimental effect on retinal microvasculature by increasing the release of inflammatory mediators, thereby further exacerbating inflammatory damage (26).

With regard to the study of lipid metabolism indicators, the present study observed significantly reduced levels of TC, LDL-C, and TG in the DR group, in contrast to the findings of some traditional studies. Two potential explanations for this discrepancy are proposed here: firstly, treatment bias. Patients in the DR group exhibited a higher prevalence of macrovascular complications, leading to a more aggressive clinical use of lipid-lowering medications such as statins and fibrates, thereby reducing lipid levels. Retrospective studies indicate that T2DM patients with DR exhibit markedly higher statin usage rates and significantly greater dosages compared to non-DR groups (27), aligning with the present hypothesis. Secondly, secondary alterations due to disease progression: prolonged severe hyperglycaemia may impair hepatic function, suppressing the activity of enzymes involved in lipid synthesis and thereby reducing hepatic lipid production. However, given that the present study did not collect data on medication use or liver metabolism-related indicators, it is not possible to verify this hypothesis. The above explanation is merely speculative and requires further confirmation in subsequent studies. A study of animals confirmed that persistent hyperglycaemia significantly reduces serum TC and LDL-C levels in mice by downregulating hepatic hydroxy-3-methylglutaryl-CoA reductase expression (28), providing experimental evidence for this mechanism. It is worth noting that NHHR levels were significantly higher in the DR group than in the non-DR group. The underlying reason for this phenomenon is that composite lipid indices provide a more accurate reflection of the core characteristics of dyslipidaemia than individual indices. Previous studies have confirmed that elevated NHHR levels reflect the accumulation of atherogenic lipoproteins. Furthermore, research has shown that elevated NHHR levels indicate impaired HDL-C-mediated reverse cholesterol transport.

Multivariate logistic regression analysis revealed that, in comparison with the Q1 group, the Q2–Q4 groups of SHR exhibited a significantly elevated risk of DR occurrence. This association remained statistically significant across four models, which adjusted for various confounding factors. The evidence suggests that even mild stress-induced hyperglycaemia may increase the risk of developing DR, providing crucial evidence for early clinical intervention. For patients with T2DM who exhibit elevated SHR, the frequency of DR screening should be increased, even when HbA1c levels are within the target range. With regard to FBG/HbA1c, the study revealed a marked elevation in DR risk across Q2–Q4 quartiles, with OR values exhibiting a progressive increase with each quartile, thereby demonstrating a discernible dose-response relationship. The dose-response relationship of FBG/HbA1c indicates that greater amplitude of glycaemic fluctuations correlates with higher DR risk. Clinicians may utilize this metric for the purpose of DR risk stratification in T2DM patients. In contrast to FBG/HbA1c, P2hBG/HbA1c exhibited a substantial risk elevation exclusively in Q3 and Q4 quartiles, with no statistical significance observed in Q2. This discrepancy may be attributable to the distinctive characteristics of 2-h postprandial glucose. At low ratios relative to HbA1c, the impact of the drug on DR may be masked by other factors; it is only when the ratio reaches a certain threshold that its driving effect on DR becomes apparent. Despite the implementation of numerous statistical analyses in this study, there exists a possibility of Type I error inflation resulting from multiple testing. As this study is exploratory in nature and no formal correction for multiple testing was performed, all significant associations should be interpreted with caution and validated in subsequent studies.

Subgroup analysis revealed that the associations between SHR, FBG/HbA1c and P2hBG/HbA1c and DR were not statistically significant across different age groups, genders, BMI categories, or subgroups defined by hypertension, coronary heart disease, stroke, PAD and MASLD. However, it should be noted that this does not imply that the association effects are entirely consistent across all subgroups; some subgroups had small sample sizes and wide confidence intervals, and the reliability of the results is limited, requiring validation in future large-scale studies. RCS analysis indicates that there is no significant non-linear relationship between the various indicators and DR, suggesting that there is a linear relationship between indicator levels and the risk of DR. In summary, the evidence suggests a strong positive relationship between indicator levels and the probability of DR occurrence. The study revealed a discrepancy in hemoglobin levels between the DR and non-DR groups. However, factors such as anemia and abnormal erythrocyte turnover have been identified as potential influencers of HbA1c test results, thereby potentially compromising the accuracy of the FBG/HbA1c and P2hBG/HbA1c ratios. This may have consequences for the interpretation of the findings of the association, and it is necessary for this to be confirmed by further investigation.

This study appears to suggest that NHHR and TG/HDL-C play a significant exploratory statistical role in the relationship between SHR, FBG/HbA1c, P2hBG/HbA1c and DR. Elevated NHHR levels are a key biomarker of dyslipidaemia, as evidenced by its role as a composite indicator integrating levels of both atherogenic and anti-atherogenic lipoproteins. In line with earlier research, SHR and glycaemic fluctuation have been shown to elevate NHHR via inflammatory and oxidative stress pathways. A body of research has demonstrated that elevated NHHR levels are associated with the onset and progression of DR through various mechanisms, including impairment of the barrier function of retinal microvascular endothelial cells, increased vascular permeability, and induction of VEGF expression (29). The TG/HDL-C ratio, a conventional marker of insulin resistance, has been demonstrated to be closely associated with elevated DR risk when elevated (30). The present study indicates that SHR and glycaemic fluctuation markers may exacerbate insulin resistance, thereby promoting hepatic TG synthesis while reducing HDL-C secretion, consequently elevating the TG/HDL-C ratio. An increased TG/HDL-C ratio has been demonstrated to exacerbate retinal microvascular lesions through a number of potential mechanisms, including the activation of inflammatory responses, the promotion of platelet aggregation, and the impairment of vascular endothelial function.

It is imperative to acknowledge the limitations of this study. Firstly, the cross-sectional design of the study does not permit the determination of temporal relationships or causality between blood glucose levels, lipid ratios and DR. Secondly, the utilization of a single-center cohort of inpatients engenders selection bias, thereby impeding the generalisability of the findings. Thirdly, the absence of data regarding medication use may result in residual confounding factors. Fourthly, the classification of DR as a binary variable obscures the existence of differences in its severity. Fifthly, the effect of hemoglobin levels on HbA1c, which is confounding, has the potential to reduce the accuracy of the glycaemic ratio. Sixthly, it is important to note that the implementation of multiple statistical tests can result in an elevated risk of Type I errors. In the seventh instance, the mediation analysis is to be regarded as exploratory research rather than as validation of biological mechanisms. Furthermore, the limited sample size in certain subgroup analyses has been identified as a factor that undermines the reliability of the results.

## Conclusion

This study indicates that SHR, FBG/HbA1c, and P2hBG/HbA1c are all positively correlated with the risk of developing DR. The present study hypothesizes that NHHR and TG/HDL-C exhibit exploratory mediating effects in these associations. However, the validity of these findings remains to be substantiated through additional research.

## Data Availability

The raw data supporting the conclusions of this article will be made available by the authors, without undue reservation.
