# Supplementary material for: Association analysis and exploratory mediation study of the stress-induced hyperglycaemia ratio and glycaemic ratio indices with diabetic retinopathy in patients with type 2 diabetes
Source: Front Nutr. 2026 May 8;13:1798444. doi: 10.3389/fnut.2026.1798444 (PMC13194131; doi:10.3389/fnut.2026.1798444)
Supplement: Supplementary file 1 [file Supplementary_file_1.docx]

Table 1. Participants demographics and baseline characteristics based on FBG/HbA1c quartiles.

| Variables | FBG/HbA1c | | | | *P* |
| --- | --- | --- | --- | --- | --- |
|  | Q1 (n = 481) | Q2 (n = 481) | Q3 (n = 481) | Q4 (n = 481) |  |
| Age (y) | 51.02 ± 15.14 | 53.50 ± 13.90 | 55.77 ± 12.66 | 57.62 ± 11.81 | <0.001 |
| Duration (y) | 2.00 (0.10,9.00) | 6.00 (1.00,10.00) | 8.00 (3.00,11.00) | 10.00 (4.00,13.00) | <0.001 |
| BMI (kg/m^2^) | 26.42 ± 3.84 | 25.67 ± 3.50 | 25.31 ± 3.40 | 24.79 ± 3.27 | <0.001 |
| SBP (mmHg) | 132.12 ± 17.14 | 131.88 ± 16.95 | 133.01 ± 17.45 | 133.27 ± 18.33 | 0.542 |
| DBP (mmHg) | 81.57 ± 12.20 | 81.01 ± 12.66 | 80.09 ± 12.76 | 78.51 ± 11.98 | <0.001 |
| FBG (mmol/L) | 5.82 ± 1.31 | 7.11 ± 1.60 | 7.97 ± 1.83 | 10.29 ± 3.05 | <0.001 |
| HbA1c (%) | 10.63 ± 2.01 | 9.50 ± 2.17 | 8.81 ± 2.01 | 8.48 ± 1.87 | <0.001 |
| SHR | 0.41 ± 0.07 | 0.58 ± 0.05 | 0.71 ± 0.06 | 0.95 ± 0.18 | <0.001 |
| P2hBG/HbA1c | 14.32 ± 4.03 | 15.96 ± 4.06 | 16.43 ± 4.35 | 18.88 ± 4.77 | <0.001 |
| BUN (mmol/L) | 6.12 ± 3.60 | 6.49 ± 3.83 | 6.64 ± 4.17 | 6.99 ± 3.08 | 0.003 |
| UA (mmol/L) | 332.00 (266.00,410.00) | 322.00 (263.00,385.00) | 321.00 (268.00,387.00) | 321.00 (258.00,385.00) | 0.130 |
| TC (mmol/L) | 4.79 (4.15,5.63) | 4.82 (4.12,5.56) | 4.75 (3.99,5.61) | 4.54 (3.79,5.29) | <0.001 |
| HDL-C (mmol/L) | 1.12 ± 0.31 | 1.15 ± 0.35 | 1.16 ± 0.27 | 1.17 ± 0.44 | 0.241 |
| LDL-C (mmol/L) | 3.01 ± 0.94 | 2.98 ± 0.89 | 2.91 ± 0.84 | 2.75 ± 0.84 | <0.001 |
| TG (mmol/L) | 1.54 (1.09,2.57) | 1.55 (1.09,2.48) | 1.55 (1.11,2.44) | 1.53 (0.96,2.36) | 0.325 |
| WBC (×10^9^/L) | 6.84 ± 3.64 | 6.89 ± 5.27 | 6.61 ± 3.53 | 6.74 ± 4.98 | 0.769 |
| Neutrophil (×10^9^/L) | 3.89 ± 2.29 | 3.68 ± 1.43 | 3.78 ± 1.96 | 3.82 ± 1.81 | 0.375 |
| Lymphocyte (×10^9^/L) | 2.19 ± 0.77 | 2.12 ± 0.72 | 2.02 ± 0.71 | 1.87 ± 0.67 | <0.001 |
| HGB (g/L) | 141.42 ± 17.19 | 139.75 ± 17.16 | 135.45 ± 17.36 | 132.16 ± 18.85 | <0.001 |
| PLT (×10^9^/L) | 207.57 ± 57.94 | 196.12 ± 55.91 | 203.65 ± 63.26 | 193.19 ± 62.12 | <0.001 |
| ALT (U/L) | 24.00 (16.00,38.00) | 22.00 (15.00,33.00) | 19.00 (14.00,29.00) | 19.00 (14.00,29.00) | <0.001 |
| AST (U/L) | 21.00 (18.00,29.00) | 20.00 (17.00,28.00) | 20.00 (16.00,26.00) | 20.00 (16.00,24.00) | <0.001 |
| Creatine (mmol/L) | 60.00 (52.00,72.00) | 60.00 (51.00,72.00) | 61.00 (50.00,72.00) | 61.00 (49.00,76.00) | 0.952 |
| Gender (male), n(%) | 347 (72.14) | 330 (68.61) | 296 (61.54) | 285 (59.25) | <0.001 |
| CHD, n(%) | 17 (3.53) | 18 (3.74) | 24 (4.99) | 27 (5.61) | 0.339 |
| Stroke, n(%) | 49 (10.19) | 48 (9.98) | 47 (9.77) | 63 (13.10) | 0.296 |
| HT, n(%) | 305 (63.41) | 276 (57.38) | 267 (55.51) | 266 (55.30) | 0.037 |
| PAD, n(%) | 160 (33.26) | 113 (23.49) | 118 (24.53) | 77 (16.01) | <0.001 |
| MASLD, n(%) | 217 (45.11) | 241 (50.10) | 253 (52.60) | 287 (59.67) | <0.001 |
| DR, n(%) | 113 (23.49) | 150 (31.19) | 180 (37.42) | 243 (50.52) | <0.001 |

Abbreviations: BMI, body mass index; SBP: systolic blood pressure; DBP: diastolic blood pressure; P2hBG: Postprandial 2-h blood glucose; HbA1c: glycated hemoglobin; WBC: white blood cell count; PLT: blood platelet count; BUN: blood urea nitrogen; Scr: scrum creatinine; UA: uric acid; TC: total cholesterol; TG: triglyceride; HDL-C: high-density lipoprotein cholesterol; LDL-C: low-density lipoprotein cholesterol; FBG: fasting blood glucose; HGB: haemoglobin; ALT: alanine aminotransferase; AST: aspartate transaminase; CHD: coronary heart disease; HT: Hypertension; PAD; Peripheral arterial disease; MASLD: Metabolic dysfunction-associated steatotic liver disease.
