# Supplementary material for: Association analysis and exploratory mediation study of the stress-induced hyperglycaemia ratio and glycaemic ratio indices with diabetic retinopathy in patients with type 2 diabetes
Source: Front Nutr. 2026 May 8;13:1798444. doi: 10.3389/fnut.2026.1798444 (PMC13194131; doi:10.3389/fnut.2026.1798444)
Supplement: Supplementary file 2 [file Supplementary_file_2.docx]

Table 2. Participants demographics and baseline characteristics based on P2hBG/HbA1c quartiles.

| Variables | P2hBG/HbA1c | | | | *P* |
| --- | --- | --- | --- | --- | --- |
|  | Q1 (n = 481) | Q2 (n = 481) | Q3 (n = 481) | Q4 (n = 481) |  |
| Age (y) | 49.88 ± 15.85 | 52.31 ± 13.52 | 56.59 ± 11.80 | 59.14 ± 11.00 | <0.001 |
| Duration (y) | 3.00 (0.10,9.00) | 4.00 (0.40,10.00) | 9.00 (3.00,11.00) | 10.00 (5.00,15.00) | <0.001 |
| BMI (kg/m^2^) | 26.99 ± 3.99 | 25.60 ± 3.24 | 25.02 ± 3.40 | 24.58 ± 3.06 | <0.001 |
| SBP (mmHg) | 132.36 ± 17.31 | 131.76 ± 17.36 | 131.90 ± 17.86 | 134.26 ± 17.29 | 0.098 |
| DBP (mmHg) | 81.76 ± 12.93 | 81.71 ± 12.49 | 79.18 ± 12.81 | 78.53 ± 11.19 | <0.001 |
| FBG (mmol/L) | 7.05 ± 2.32 | 7.67 ± 2.59 | 8.01 ± 2.50 | 8.47 ± 2.86 | <0.001 |
| HbA1c (%) | 10.66 ± 2.22 | 10.00 ± 2.02 | 8.86 ± 1.80 | 7.90 ± 1.50 | <0.001 |
| FBG/HbA1c | 12.13 ± 3.68 | 13.95 ± 4.11 | 16.25 ± 3.60 | 19.21 ± 5.01 | <0.001 |
| SHR | 0.51 ± 0.16 | 0.59 ± 0.18 | 0.70 ± 0.16 | 0.85 ± 0.22 | <0.001 |
| BUN (mmol/L) | 6.23 ± 4.73 | 6.33 ± 2.55 | 6.75 ± 2.75 | 6.94 ± 4.26 | 0.007 |
| UA (mmol/L) | 339.00 (270.00,422.00) | 323.00 (266.00,390.00) | 321.00 (256.00,385.00) | 312.00 (258.00,380.00) | <0.001 |
| TC (mmol/L) | 4.80 (4.17,5.65) | 4.88 (4.24,5.67) | 4.71 (3.95,5.40) | 4.44 (3.71,5.33) | <0.001 |
| HDL-C (mmol/L) | 1.11 ± 0.29 | 1.13 ± 0.35 | 1.16 ± 0.32 | 1.21 ± 0.42 | 0.160 |
| LDL-C (mmol/L) | 3.04 ± 0.91 | 3.01 ± 0.89 | 2.87 ± 0.83 | 2.72 ± 0.86 | <0.001 |
| TG (mmol/L) | 1.71 (1.22,2.86) | 1.67 (1.14,2.78) | 1.50 (1.08,2.37) | 1.33 (0.92,2.00) | <0.001 |
| WBC (×10^9^/L) | 7.23 ± 3.98 | 6.78 ± 4.58 | 6.73 ± 4.96 | 6.35 ± 4.06 | 0.021 |
| Neutrophil (×10^9^/L) | 4.02 ± 1.69 | 3.80 ± 2.32 | 3.80 ± 1.98 | 3.56 ± 1.48 | 0.002 |
| Lymphocyte (×10^9^/L) | 2.26 ± 0.78 | 2.09 ± 0.70 | 1.96 ± 0.68 | 1.88 ± 0.69 | <0.001 |
| HGB (g/L) | 141.48 ± 16.28 | 139.37 ± 17.90 | 135.50 ± 17.20 | 132.43 ± 19.22 | <0.001 |
| PLT (×10^9^/L) | 212.13 ± 58.34 | 203.09 ± 58.62 | 195.56 ± 60.78 | 189.74 ± 60.50 | <0.001 |
| ALT (U/L) | 25.00 (17.00,40.00) | 22.00 (16.00,35.00) | 19.00 (15.00,29.00) | 19.00 (13.00,28.00) | <0.001 |
| AST (U/L) | 21.00 (18.00,29.00) | 20.00 (17.00,27.00) | 20.00 (16.00,26.00) | 19.00 (16.00,24.00) | <0.001 |
| Creatine (mmol/L) | 60.00 (51.00,72.00) | 61.00 (51.00,72.00) | 61.00 (50.00,72.00) | 61.00 (50.00,75.00) | 0.795 |
| Gender (male), n(%) | 334 (69.44) | 331 (68.81) | 309 (64.24) | 284 (59.04) | 0.002 |
| CHD, n(%) | 18 (3.74) | 14 (2.91) | 25 (5.20) | 29 (6.03) | 0.083 |
| Stroke, n(%) | 45 (9.36) | 48 (9.98) | 52 (10.81) | 62 (12.89) | 0.312 |
| HT, n(%) | 303 (62.99) | 289 (60.08) | 278 (57.80) | 244 (50.73) | 0.001 |
| PAD, n(%) | 176 (36.59) | 128 (26.61) | 88 (18.30) | 76 (15.80) | <0.001 |
| MASLD, n(%) | 201 (41.79) | 234 (48.65) | 266 (55.30) | 297 (61.75) | <0.001 |
| DR, n(%) | 139 (28.90) | 162 (33.68) | 178 (37.01) | 207 (43.04) | <0.001 |

Abbreviations: BMI, body mass index; SBP: systolic blood pressure; DBP: diastolic blood pressure; P2hBG: Postprandial 2-h blood glucose; HbA1c: glycated hemoglobin; WBC: white blood cell count; PLT: blood platelet count; BUN: blood urea nitrogen; Scr: scrum creatinine; UA: uric acid; TC: total cholesterol; TG: triglyceride; HDL-C: high-density lipoprotein cholesterol; LDL-C: low-density lipoprotein cholesterol; FBG: fasting blood glucose; HGB: haemoglobin; ALT: alanine aminotransferase; AST: aspartate transaminase; CHD: coronary heart disease; HT: Hypertension; PAD; Peripheral arterial disease; MASLD: Metabolic dysfunction-associated steatotic liver disease.
